# Dependence of the hindgut disappearance of phosphorus in pigs on the quantity of phosphorus entering the hindgut based on a Meta-analysis

**DOI:** 10.1016/j.aninu.2024.07.007

**Published:** 2024-10-05

**Authors:** Noa Park, Beob Gyun Kim

**Affiliations:** Department of Animal Science, Konkuk University, Seoul 05029, Republic of Korea

**Keywords:** Hindgut disappearance, Meta-analysis, Phosphorus, Swine

## Abstract

The objectives of the current study were to compare the difference between standardized ileal digestibility (SID) and standardized total tract digestibility (STTD) of phosphorus (P) in pigs using published data and investigate the factors that affect the hindgut disappearance of P in pigs. A total of 156 observations from 32 experiments that determined the apparent ileal digestibility and total tract digestibility of P in pigs were collected. The SID and STTD of P were calculated by accounting for basal endogenous losses of P. Standardized hindgut disappearance (SHD) of P was determined by subtracting the SID of P from the STTD of P. The Chi-square test was performed to investigate the association between SHD of P and categorical variables, including the use of phytase, the use of inorganic P sources, the use of corn-soybean meal-based diets, and body weight (BW) of pigs. To determine the effects of the SID of P on the SHD of P, a linear equation for the SHD of P was developed using the SID of P as an independent variable. The BW of pigs ranged from 10.0 to 104.8 kg and the SHD of P ranged from −22.8% to 39.8%. The STTD of P was greater than the SID of P (47.1% vs. 49.7%; *P* = 0.019). Based on the Chi-square analysis, the supplementation of inorganic P sources tended to result in a higher occurrence of a positive value for the SHD of P (*P* = 0.079). In addition, the occurrence of a positive value in the SHD of P was lower when the BW of pigs was below 30 kg. However, as the BW of pigs increased, the occurrence of a positive value in the SHD of P increased (*P* = 0.061). A regression analysis of the SHD of P against the SID of P in pigs indicated that the SHD of P decreased as the SID of P increased in pigs (*r*^2^ = 0.17; *P* < 0.001). In conclusion, the STTD of P is greater than the SID of P in pigs, and the SHD of P depends on the diet composition, the amount of P entering the large intestine, and the BW of the pigs.

## 1 Introduction

As phosphorus (P) is an essential inorganic nutrient for pigs playing a crucial role in various biological processes and bone growth, providing pigs with a sufficient quantity of P is essential. However, due to the high cost of P sources and concerns about environmental pollution (Han et al., 2001), P concentrations in swine diets should be precisely formulated to meet the requirements. Numerous studies have been conducted to accurately determine the P requirement and digestibility in pigs (Adeola et al., 2015; Hong and Kim, 2021; Kong et al., 2021). Traditionally, the availability of P has been determined using the relative bioavailability of P (Cromwell, 1992; NRC, 1998). However, relative bioavailability values have limitations for feed formulation due to non-additivity in mixed diets (NRC, 2012). Therefore, the concept of a standardized digestibility assay for P has been adopted, similarly to amino acids (NRC, 2012), allowing for a more precise estimation of P utilization in swine nutrition.

With the adoption of the standardized digestibility assay, P digestibility has been evaluated at the total tract level because it is easier to do so for determining P digestibility than at the ileal level (Lee and Stein, 2023). Several studies have shown no difference between the ileal digestibility and the total tract digestibility of P in pigs (Dilger and Adeola, 2006; Fan et al., 2001; Zhang et al., 2016). Furthermore, Jongbloed et al. (1992) documented even negative hindgut disappearance (HD) of P in pigs. Consequently, the hindgut absorption of P has often been considered negligible in pigs. However, some studies have reported the presence of HD of P, which is calculated by subtracting the ileal digestibility value from the total tract digestibility value, under certain dietary conditions or physiological stages of pigs. When pigs are fed a diet with a low calcium (Ca)-to-P ratio (Ca:P; Liu et al., 2000) or a corn-soybean meal (SBM)-based diet (Liu et al., 2017), the positive HD of P has been reported. Similarly, positive HD of P in pigs was observed when pigs reached a body weight (BW) greater than 60 kg (Liu et al., 2018). These inconsistent findings regarding the HD of P suggest that the large intestine may have the ability to utilize P under specific environmental conditions. Nevertheless, information on HD of P is limited due to the difficulties in its determination and interpretation (Son et al., 2023). Therefore, the present study aimed to investigate the difference between the ileal digestibility and the total tract digestibility of P in pigs using published data and investigate the factors that could affect HD of P in pigs with a hypothesis that the total tract digestibility of P is greater than ileal digestibility of P in pigs.

## 2 Materials and methods

### 2.1 Data collection and processing

A dataset was compiled by searching online databases at Google Scholar based on the following keywords: pig, phosphorus, ileal digestibility, total tract digestibility, hindgut disappearance, and swine. The studies obtained from the literature search were then screened manually based on their titles and experimental information. Data on ingredient composition, dietary contents of amylase-treated neutral detergent fiber (aNDF), P, phytate-P, Ca, and dry matter (DM), and BW of pigs were collected. Additionally, values for apparent ileal digestibility (AID) and apparent total tract digestibility (ATTD) of P, the number of observations, and standard error of the means were also collected. A total of 156 observations from 32 experiments in 26 literature sources, which determined the AID and ATTD of P in pigs, were included in the dataset (Table 1). Each observation represents a mean value presented in the research article. In the collected literature, the index method was used to determine AID and ATTD of P in feed ingredients and diets for pigs as described by Kong and Adeola (2014), and the ileal and total tract digestibility of P was measured in the same pigs that were equipped with ileal cannulas and individually housed. Based on the values of AID and ATTD of P, standardized ileal digestibility (SID) and standardized total tract digestibility (STTD) of P were calculated by correcting basal endogenous losses of P. Additionally, the apparent hindgut disappearance (AHD) and standardized hindgut disappearance (SHD) of P were also calculated. In some experiments, the DM concentration (89 observations from 16 studies), aNDF concentration (125 observations from 18 studies), and phytate-P concentration (95 observations from 17 studies) of the diets were not provided. In such cases, the DM concentration of diets was assumed to be 90% and aNDF and phytate-P concentrations in the diets were calculated based on the ingredient composition of the experimental diets and the feed tables in the NRC (2012).

**Table 1:** Variability of body weight of pigs, total calcium (Ca), phosphorus (P), phytate-P, ileal digestibility, total tract digestibility, and hindgut disappearance of P in the literature (dry matter basis)^1^.

Item	Arithmetic mean	Minimum	Maximum	Standard deviation
Body weight, kg	41.2	10.0	104.8	20.6
aNDF, %	11.9	1.6	32.6	4.9
Total Ca, %	0.64	0.01	1.13	0.25
Total P, %	0.52	0.09	0.91	0.17
Ca:P	1.27	0.04	3.20	0.49
Phytate-P, %	0.28	0.02	0.66	0.13
AID of P, %	42.2	7.2	75.9	14.1
ATTD of P, %	45.8	15.0	78.0	12.6
AHD of P, %	3.6	−23.1	39.6	7.7
SID of P^2^, %	46.2	9.4	80.1	14.4
STTD of P^2^, %	50.1	20.3	82.5	12.6
SHD of P, %	3.9	−22.8	39.8	7.7

aNDF = amylase-treated neutral detergent fiber; AID = apparent ileal digestibility; ATTD = apparent total tract digestibility; AHD = apparent hindgut disappearance; SID = standardized ileal digestibility; STTD = standardized total tract digestibility; SHD = standardized hindgut disappearance.

1 The values were based on 32 experiments from 26 research papers (*n* = 156; Baumgärtel et al., 2008; Blaabjerg et al., 2010; Bohlke et al., 2005; Bruce and Sundstøl, 1995; Dilger and Adeola, 2006; Espinosa et al., 2022; Fan and Sauer, 2002; Ginste and De Schrijver, 1998; Heyer et al., 2022; Johnston et al., 2004; Li et al., 1999; Lindberg et al., 2007; Liu et al., 2017, 2018; Mok et al., 2013; Nortey et al., 2008, 2007; Rutherfurd et al., 2014; Sands et al., 2009; Son et al., 2023; Steiner et al., 2006; Tian et al., 2023; Valaja et al., 1998; Yang et al., 2020; Yáñez et al., 2011; Zhang et al., 2016).

2 The SID and STTD of P were calculated by correcting the AID and ATTD of P for ileal basal endogenous losses of P (177 mg/kg dry matter intake; Traylor et al., 2001) and total tract basal endogenous losses of P (190 mg/kg dry matter intake; NRC, 2012), respectively.

### 2.2 Calculations

The SID or STTD of P was calculated using the following equation (Sung and Kim, 2019):

SID or STTD of P (%) = AID or ATTD of P + [(basal endogenous losses of P/P concentration in diet) × 100],

where the basal endogenous losses of P at the ileal level were considered to be 177 mg/kg DM intake as described by Traylor et al. (2001). At the total tract level, the basal endogenous losses of P were considered to be 190 mg/kg DM intake as specified by the NRC (2012).

The HD of P was calculated using the following equations (Son et al., 2023):

AHD of P (%) = ATTD of P (%) – AID of P (%);

SHD of P (%) = STTD of P (%) – SID of P (%).

The standardized ileal digestible P, ileal undigestible P, total tract digestible P, total tract undigestible P, hindgut digestible P, and hindgut undigestible P were calculated using the following equations (Liu et al., 2017):

Standardized ileal digestible P (g/d) = P intake (g/d) × SID (%);

Standardized ileal undigestible P (g/d) = P intake (g/d) – standardized ileal digestible P (g/d);

Standardized total tract digestible P (g/d) = P intake (g/d) × STTD (%);

Standardized hindgut digestible P (g/d) = standardized total tract digestible P (g/d) – standardized ileal digestible P (g/d);

Standardized hindgut undigestible P (g/d) = standardized ileal digestible P (g/d) – standardized hindgut digestible P (g/d).

### 2.3 Statistical analysis

The observations for each digestibility value were analyzed using the MIXED procedure of SAS 9.4 (SAS Inst. Inc., Cary, NC, USA). The apparent and standardized digestibility data were weighted using the WEIGHT statement of SAS, where the weights were the inverse of squared standard error of the means. The model for the comparison of the digestibility values of P was:

*Y*_*ij*_ = *μ* + method_*i*_ + experiment_*j*_ + *ω*_*j*_ + *ε*_*ij*_;

where *Yij* is the response variable, *μ* is the overall mean, *ω*_*j*_ is the weight, and *ε*_*ij*_ is the residual error. The method (*i* = 1 for ileal digestibility and 2 for total tract digestibility) for digestibility assessment was considered a fixed variable, and experiment (*j* = 1–32) was considered a random variable. The level at which the digestibility was determined was considered a fixed variable, whereas the experiment was regarded as a random variable. The Chi-square test was performed to investigate the association between HD of P and categorical variables, including the use of phytase, the use of inorganic P sources, the use of corn-SBM-based diets, and the BW of pigs. When the degree of freedom of cross-tabulation was 1, a Yates correction was applied (Yates, 1984). The experimental unit was a diet, and the significance and tendency of treatment effects were declared at *P* < 0.05 and 0.05 ≤ *P* < 0.10, respectively. Furthermore, a simple regression analysis was conducted by the REG procedure of SAS with the WEIGHT statement to verify linear relationships among the variables. In the REG procedure, the weights were the inverse of squared standard error of the means.

## 3 Results

The initial BW of pigs ranged from 10.0 to 104.8 kg (Table 1). The diets used in the present study had total P concentrations ranging from 0.09% to 0.91% and phytate-P concentrations from 0.02% to 0.66% on a DM basis. The AHD and SHD of P varied from −23.1% to 39.6% and −22.8% to 39.8%, respectively.

The ATTD of P was greater than the AID of P (*P* = 0.035; Table 2). Additionally, the STTD of P was greater than the SID of P (*P* = 0.019). The AHD of P was positive in 116 out of 156 observations (Table 3). The Chi-square analysis indicated that the supplementation of inorganic P sources tended to result in a higher occurrence of a positive value for AHD of P (*P* = 0.099). In addition, there was a relationship between the AHD of P and BW of pigs (*P* = 0.027). When the BW of pigs was below 30 kg, the occurrence of positive AHD of P was lower. However, as the BW of pigs increased, the occurrence of positive AHD of P increased.

**Table 2:** Comparison between ileal digestibility and total tract digestibility (%) of phosphorus in pigs (*n* = 156)^1^.

Item	Ileal digestibility	Total tract digestibility	SEM	*P*-value
Apparent	43.2	45.6	1.90	0.035
Standardized	47.1	49.7	1.94	0.019

1 The digestibility of phosphorus was calculated as weighted mean based on the inverse of the standard error of the mean squared.

**Table 3:** A Chi-square analysis of apparent hindgut disappearance (AHD) of phosphorus^1^.

Item	AHD <0	AHD >0	Chi-square	*P*-value
The number of observations	40	116		
Use of phytase^2^, ^3^			0.01	0.936
Used	18 (19)^4^	55 (54)		
Not used	22 (21)	61 (62)		
Use of inorganic phosphorus sources^2^			2.72	0.099
Used	11 (16)	51 (46)		
Not used	29 (24)	65 (70)		
Corn-soybean meal-based^2^			0.51	0.478
Used	17 (14)	39 (42)		
Not used	23 (26)	77 (74)		
BW, kg			9.17	0.027
0 < BW < 30	19 (13)	33 (39)		
30 ≤ BW < 45	7 (12)	39 (34)		
45 ≤ BW < 60	5 (8)	28 (25)		
60 ≤ BW < 105	9 (6)	16 (19)		

BW = body weight.

1 Apparent hindgut disappearance was the deviation between apparent total tract digestibility and apparent ileal digestibility.

2 Chi-square analysis was performed with Yates correction.

3 The number of phytase use includes the use of phytase, wheat, or wheat byproduct.

4 Numbers in parenthesis represent expected value.

Similarly, the SHD of P was positive in 118 observations out of 156 total data (Table 4). Based on the Chi-square analysis, the supplementation of inorganic P sources tended to result in a higher occurrence of a positive value for SHD of P (*P* = 0.079). The BW of pigs also tended to be associated with the occurrence of positive value in SHD of P (*P* = 0.061) in that the occurrence of positive value in SHD of P was lower when the BW of pigs were below 30 kg whereas the occurrence of positive value in SHD of P increased as the BW of pigs increased at over 30 kg.

**Table 4:** A Chi-square analysis of standardized hindgut disappearance (SHD) of phosphorus^1^.

Item	SHD <0	SHD >0	Chi-square	*P*-value
The number of observations	38	118		
Use of phytase^2^, ^3^			0.01	0.916
Used	17 (18)^4^	56 (55)		
Not used	21 (20)	62 (63)		
Use of inorganic phosphorus sources^2^			3.08	0.079
Used	10 (15)	52 (47)		
Not used	28 (23)	66 (71)		
Corn-soybean meal-based^2^			0.52	0.470
Used	16 (14)	40 (42)		
Not used	22 (24)	78 (76)		
BW, kg			7.37	0.061
0 < BW < 30	18 (13)	34 (39)		
30 ≤ BW < 45	7 (11)	39 (35)		
45 ≤ BW < 60	5 (8)	28 (25)		
60 ≤ BW < 105	8 (6)	17 (19)		

BW = body weight.

1 Standardized hindgut disappearance was the deviation between standardized total tract digestibility and standardized ileal digestibility.

2 Chi-square analysis was performed with Yates correction.

3 The number of phytase use includes the use of phytase, wheat, or wheat byproduct.

4 Numbers in parenthesis represent expected value.

**Fig. 1:**
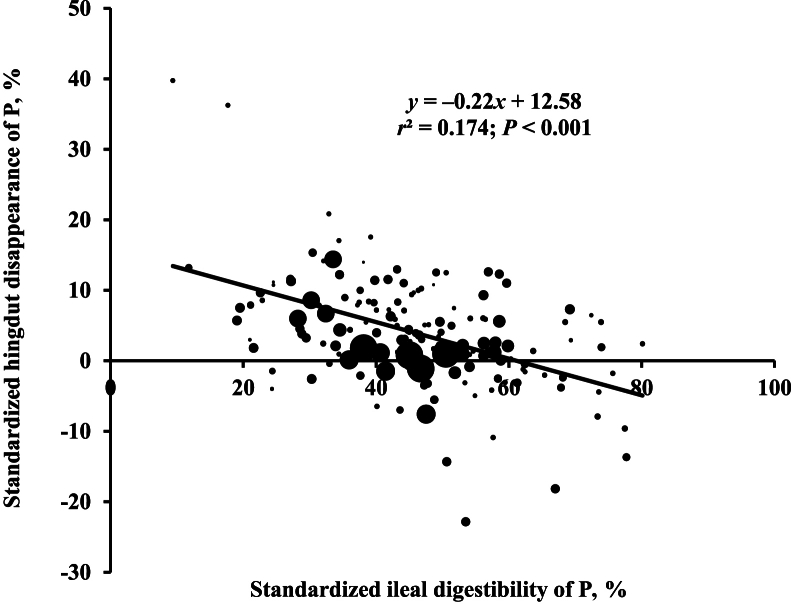
Relationship between standardized hindgut disappearance (SHD) of phosphorus (P) and standardized ileal digestibility of P in pigs (*n* = 156). The bubble size represents the weight of each observation. The weight was the inverse of squared standard error of the means. The SHD of P was calculated by subtracting the standardized ileal digestibility of P from the standardized total tract digestibility of P of pigs.

**Fig. 2:**
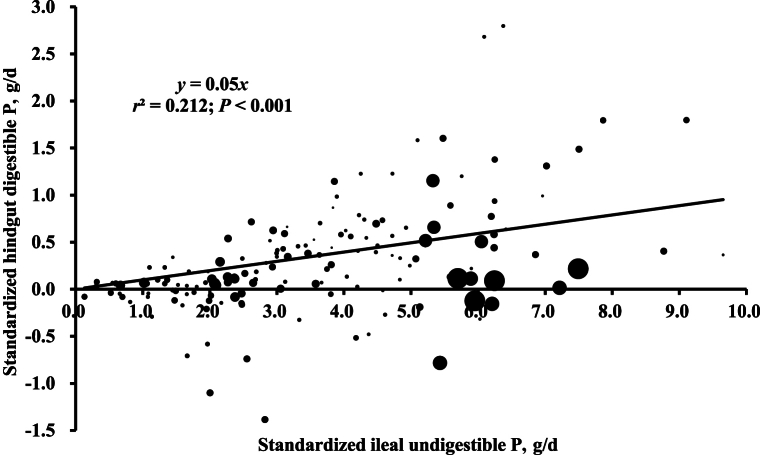
Relationship between daily amounts of standardized ileal undigestible phosphorus (P, g/d) and standardized hindgut digestible P (g/d) in pigs (*n* = 156). The standardized ileal undigestible P was calculated by subtracting the standardized ileal digestible P (g/d) from P intake (g/d). The bubble size represents the weight of each observation. The weight was the inverse of squared standard error of the means. The Y-intercept was forced to zero due to the lack of significant intercept.

**Fig. 3:**
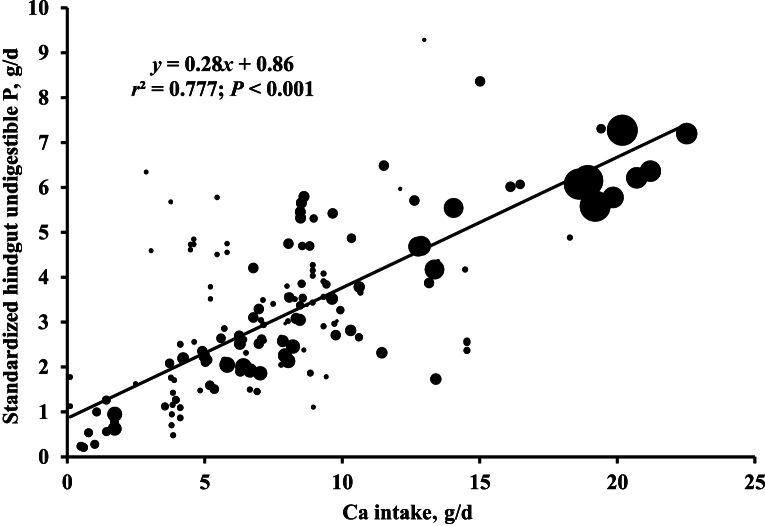
Relationship between daily amounts of calcium intake (g/d) and standardized hindgut undigestible phosphorus (P, g/d) in pigs (*n* = 156). The bubble size represents the weight of each observation. The weight was the inverse of squared standard error of the means. The standardized hindgut undigestible P was calculated by subtracting the standardized hindgut digestible P (g/d) from standardized ileal digestible P (g/d).

Regression analyses of the SHD of P against SID of P in pigs indicated that the SHD of P decreased as the SID of P increased in pigs (Fig. 1; *r*^2^ = 0.174). The SHD of P was increased with standardized ileal undigestible P when expressed as g/d (Fig. 2; *r*^2^ = 0.212). The standardized hindgut undigestible P increased with Ca intake when expressed as g/d (Fig. 3; *r*^2^ = 0.777).

## 4 Discussion

To determine standardized P digestibility, the total tract digestibility has been employed due to its practicality and cost-effectiveness compared with the ileal digestibility (Lee and Stein, 2023). Several studies reported no difference between ileal and total tract digestibility of P in pigs (Dilger and Adeola, 2006; Fan et al., 2001; Zhang et al., 2016), and thus, the total tract digestibility has been used with the assumption of equivalence between ileal and total tract digestibility of P in pigs. Consequently, the contribution of the large intestine to P digestion and absorption in pigs has been traditionally considered negligible. However, other studies have reported the existence of absorption of P in the large intestine of pigs. Liu et al. (2000) reported that P was absorbed in the large intestine of 123-kg pigs fed corn-SBM-based diets. Similarly, Son et al. (2023) observed a 10.5% AHD of P when pigs were fed a wheat-SBM-based diet containing 20% wheat bran and dicalcium phosphate at 0.1%, although no linear or quadratic effects of wheat bran on AHD of P were observed. Fan et al. (2001) proposed that the absorption of P in the large intestine of pigs primarily involves reabsorption of endogenous losses of P, rather than ingested P, resulting in zero P retention in the large intestine. Despite numerous studies on P utilization in the hindgut of pigs, controversies still exist (Liu et al., 2000, 2017; Son et al., 2023). In the present study, the greater STTD than SID of P in pigs was observed, consistent with previous findings (Johnston et al., 2004; Liu et al., 2017, 2018), suggesting net absorption of P in the hindgut of pigs.

The varied results of interactions between the use of phytase or the use of inorganic P sources and the HD of P were unexpected (Table 3, Table 4), considering that both phytase and inorganic P sources would result in a reduced amount of undigested P entering the hindgut of pigs. Supplemented phytase primarily hydrolyzes phytate-P in the stomach and proximal small intestine before reaching the jejunum (Park and Adeola, 2023). The phosphate liberated from phytate by supplemented phytase is readily absorbed before reaching the end of the ileum through passive diffusion via tight junctions and active transport through sodium-dependent phosphate transporters in the small intestine (Sabbagh et al., 2011). Likewise, when inorganic P sources are supplemented, the inorganic P sources are solubilized by the high acidity in the stomach and are readily absorbed in the small intestine (Goss et al., 2007). Therefore, the digestion and absorption of P in diets with relatively high inorganic P concentrations, in comparison to diets lacking inorganic P sources, are completed before reaching the end of the ileum, as is the case with phytase supplementation (Zhai et al., 2022). This leads to relatively small amounts of undigested P entering the hindgut of pigs. Consequently, the HD of P may be restricted when either phytase or inorganic P sources are used, as the HD of P depends on the quantity of undigested P entering the hindgut. However, the results of the Chi-square tests were not consistent with the linear relationships between the SHD of P and the SID of P or standardized hindgut undigested P in the present study. Although the rationale for this discrepancy remains unclear, this could be attributed to the classification criterion, which depends on the presence or absence of phytase or an inorganic P source rather than their concentration levels, based on the dichotomous characteristics of the Chi-square analysis. For instance, even if there is a small amount of phytase and inorganic P source enough to exhibit a positive HD of P in the diet, it can be classified as the presence of phytase or an inorganic P source in the Chi-square test. Alternatively, the inconsistency between the regression analyses and the Chi-square tests could be partially explained by different diet constituents such as the concentration of phytate or the physiological conditions of pigs.

Several studies showed that phytase or inorganic P supplementation reduces the HD of P. Johnston et al. (2004) reported that AHD of P decreased from 9.1% to 5.8% when pigs with a BW of 53 kg were fed a diet supplemented with phytase and monocalcium phosphate as an inorganic P source. Furthermore, Bruce and Sundstøl (1995) reported that the diet with both monocalcium phosphate at 0.55% and phytase at 375 FTU/kg had less AHD of P (2.7%) compared with the diets with phytase at 750 FTU/kg (AHD of P = 6.3%), the diet with monocalcium phosphate at 1.11% (AHD of P = 7.3%), or the diets without inorganic P source or phytase (AHD of P = 9.4%). However, Mok et al. (2013) reported that there was no significant difference when comparing pigs fed a corn-SBM-palm kernel meal-based diet supplemented with phytase at 1000 FTU/kg and those without phytase supplementation (9.2% vs. 12.1%). Additionally, when 27.8-kg pigs were fed a corn-SBM-based diet with increasing dicalcium phosphate, no response of AHD of P was observed (Liu et al., 2017). Therefore, inconsistency in the magnitude of supplementation of phytase or inorganic P sources in diets among the published studies necessitates further research to elucidate on the impact of phytase and inorganic P source supplementation on the extent of HD of P in pigs.

The observed interaction between BW of pigs and HD of P can be explained by the findings of Liu et al. (2018), who demonstrated that pigs above 60 kg BW exhibited HD of P due to their greater capacity for hindgut digestion and absorption of P compared with pigs weighing 25 kg. This can be attributed to the increasing length of the large intestine in pigs as their BW increases, resulting in a longer residence time of digesta, and thus, the potential of absorption by the host or microbial fermentation in the large intestine may increase (Noblet and Shi, 1993, 1994). Finishing pigs have a greater potential for nutrient digestion and absorption or fermentation due to their longer gastrointestinal length and retention time compared with nursery or growing pigs (Noblet and Shi, 1994). In this manner, pigs with higher BW may exhibit a greater digestion capacity for P in the hindgut, leading to greater absorption or fermentation in the hindgut.

Moreover, the HD of P in pigs, which is dependent on BW, may be influenced by the diet composition. During the nursery stage of pigs, it is common to use animal-derived feed ingredients that contain greater amount of digestible P compared with P sources originated from plants (Kong et al., 2021). Phosphorus in animal sources is highly digestible compared with plant-derived P and may be readily absorbed before reaching the end of the ileum, which can result in low HD of P. In such cases, the HD of P may be limited regardless of the length of the gastrointestinal tract. On the other hand, the inclusion of fibrous sources such as wheat bran or palm kernel meal in the diet during the growing stage of pigs is common and may lower the digestibility of P until the ileum due to the shorter transit time in the gut due to the increased peristalsis by fiber fraction (Nortey et al., 2007). This can lead to a larger amount of P entering the hindgut, potentially resulting in the occurrence of HD of P. However, the influence of dietary fiber on the amount or proportion of P entering the large intestine was not observed in the present study. In the older pigs with a greater daily feed intake, the increased P intake can also lead to a larger amount of P entering the hindgut, thereby increasing the occurrence of HD of P (Liu et al., 2018).

Calcium may influence the HD of P in pigs. It is widely recognized that an excessive dietary Ca concentration or high Ca:P in diets can lead to reduced P digestibility in pigs (Klein et al., 2023; Lee et al., 2020; Stein et al., 2011). Despite the variations in feeds used in this meta-analysis, a strong linear relationship between Ca intake and standardized hindgut undigested P was observed. This suggests that large amount of Ca intake disrupts P absorption, extending this effect to the hindgut of pigs. Previous studies by Stein et al. (2011) and Lee et al. (2020) reported that increased Ca intake in the form of Ca carbonate resulted in a linear reduction in the ATTD of P in growing pigs and gestating sows, respectively. These studies discussed the potential for Ca to bind with P in the gastrointestinal tract of pigs, forming a Ca–P complex that is less readily digested and absorbed. Although this phenomenon has not been fully elucidated, it can be partially explained by the lower solubility of Ca–P complexes, such as Ca phosphate, in the lower gastrointestinal tract of pigs (Lee et al., 2020; Stein et al., 2011). Whereas Ca is mainly absorbed in the duodenum, P is mainly absorbed in both the duodenum and jejunum in pigs (Hoag and Dharmarajan, 2021). If excessive Ca is included in the diets, the unabsorbed inorganic form of P, phosphate, may combine with undigested Ca, resulting in the formation of a Ca-phosphate complex in the jejunum and ileum of pigs. Because the solubility of Ca-phosphate complex is maximized at a pH less than 3.0 such as the stomach, the solubility of Ca-phosphate complex sharply increased as the pH decreased from 6.0 to 2.0 (Goss et al., 2007). Because the pH in the duodenum, the jejunum to ileum, and the cecum to colon is from 3.1 to 5.5, from 5.6 to 6.0, and from 6.1 to 7.0, respectively (Roura et al., 2023), when Ca absorption ends in the duodenum, undigested Ca and P may form a Ca–P complex, and the solubility of Ca–P complex is further decreased due to the relatively high pH in the terminal small intestine and in the large intestine of pigs. This inference is supported by the results of Hoag and Dharmarajan (2021) who reported that Ca-salts inhibit P absorption, and therefore, a high Ca intake has the potential to bind P in the gastrointestinal tract. Moreover, it is also consistent with the results of increased absorption in the cecum and colon of pigs when the Ca:P is decreased from 1.5 to 1.0 in diets (Liu et al., 2000) and increased AHD of P when the ratio of Ca-to-digestible P decreased from 3:1 to 2:1 with decreasing limestone (Klein et al., 2023). As a result, the HD of P in pigs can be decreased or limited when a high Ca concentration is included in the diet.

Based on the findings in the present meta-analysis, the absorption of P in the large intestine is revealed. Smith et al. (1955) first suggested this phenomenon, identifying the cecum and colon as potential sites for regulating P homeostasis in pigs by observing the absorption of endogenous P losses using radioactive isotope P. Although the metabolic processes of P in the cecum and colon remain unclear, Seynaeve et al. (2000) suggested that phytates entering the hindgut are almost completely hydrolyzed by microbial enzymes allowing potential absorption of P in the cecum and the colon. For the absorption mechanism in the large intestine, Wubuli et al. (2019) reported the expression of genes for sodium-dependent phosphate transporters in the cecum and colon of 100-kg pigs, indicating that P can be transported from the lumen of the large intestine to the circulatory system. Although not in pigs, a sodium-dependent phosphate transporter was observed in the colon of rats (He et al., 2016). Further research is warranted to identify phosphate transporters in the hindgut of pigs.

In the present analysis, the ATTD of P was greater than the AID of P in 116 observations whereas the ATTD of P was less than the AID of P in 40 observations. In an extreme case for the negative HD of P, ATTD of P was 19 percentage unit less than AID of P in 30-kg pigs fed corn-SBM-based diets (Yang et al., 2020), indicating that a large quantity of endogenous P was secreted in the hindgut but the absorption of P in the hindgut was minimal. The specific reasons for negative HD of P remain unclear. However, Fan et al. (2001) suggested that a large portion of endogenous losses of P is reabsorbed in the large intestine. Moreover, the endogenous losses of P in the hindgut are relatively small considering that the basal endogenous losses of P at the end of the ileum are 177 mg/kg DM intake (Traylor et al., 2001) and those in the total tract are 190 mg/kg DM intake (NRC, 2012).

## 5 Conclusions

The present work determined hindgut disappearance of P for pigs using a Meta-analysis. The hindgut disappearance of P in pigs exists and is influenced by factors including the diet composition, the amount of P entering the large intestine, and the body weight. The determination of P digestibility at the total tract level may be appropriate for an accurate evaluation of P digestibility. Further research is warranted to investigate the metabolism of P in the large intestine of pigs.

## Credit Author Statement

**Noa Park:** investigation, conceptualization, methodology, formal analysis, writing – original draft; **Beob Gyun Kim:** conceptualization, supervision, writing – review & editing.

## Declaration of competing interest

We declare that we have no financial and personal relationships with other people or organizations that can inappropriately influence our work, and there is no professional or other personal interest of any nature or kind in any product, service and/or company that could be construed as influencing the content of this paper.
